# Altered cerebral hemodyamics and cortical thinning in asymptomatic carotid artery stenosis

**DOI:** 10.1371/journal.pone.0189727

**Published:** 2017-12-14

**Authors:** Randolph S. Marshall, Iris Asllani, Marykay A. Pavol, Ying-Kuen Cheung, Ronald M. Lazar

**Affiliations:** 1 Department of Neurology, Columbia University Medical Center, New York, New York, United States of America; 2 Department of Biomedical Engineering, Rochester Institute of Technology, College of Engineering, Rochester, New York, United States of America; Banner Alzheimer's Institute, UNITED STATES

## Abstract

Cortical thinning is a potentially important biomarker, but the pathophysiology in cerebrovascular disease is unknown. We investigated the association between regional cortical blood flow and regional cortical thickness in patients with asymptomatic unilateral high-grade internal carotid artery disease without stroke. Twenty-nine patients underwent high resolution anatomical and single-delay, pseudocontinuous arterial spin labeling magnetic resonance imaging with partial volume correction to assess gray matter baseline flow. Cortical thickness was estimated using Freesurfer software, followed by co-registration onto each patient’s cerebral blood flow image space. Paired t-tests assessed regional cerebral blood flow in motor cortex (supplied by the carotid artery) and visual cortex (indirectly supplied by the carotid) on the occluded and unoccluded side. Pearson correlations were calculated between cortical thickness and regional cerebral blood flow, along with age, hypertension, diabetes and white matter hyperintensity volume. Multiple regression and generalized estimating equation were used to predict cortical thickness bilaterally and in each hemisphere separately. Cortical blood flow correlated with thickness in motor cortex bilaterally (p = 0.0002), and in the occluded and unoccluded sides individually; age (p = 0.002) was also a predictor of cortical thickness in the motor cortex. None of the variables predicted cortical thickness in visual cortex. Blood flow was significantly lower on the occluded versus unoccluded side in the motor cortex (p<0.0001) and in the visual cortex (p = 0.018). On average, cortex was thinner on the side of occlusion in motor but not in visual cortex. The association between cortical blood flow and cortical thickness in carotid arterial territory with greater thinning on the side of the carotid occlusion suggests that altered cerebral hemodynamics is a factor in cortical thinning.

## Introduction

Thinning of the cortex in the brain has been linked to cognitive impairment in neurodegenerative dementias[1–4] and vascular disease[5–7], making the radiographic assessment of cortical thickness a potentially valuable biomarker for cognitive decline.[8] It has been difficult to determine the underlying causal mechanisms of cortical thinning, however, which is likely to differ by disease type. In patients with cardiovascular disease, it has been reported that chronic hypertension[9], diabetes[10, 11] and heart failure[12] confer risk for generalized cortical thinning. In addition, cortical thinning has been associated with clinically and radiographically apparent cerebral ischemic injury, both in small-vessel and large-vessel infarction, and in association with white matter hyperintensity volumes (WMHV).[13, 14] Yet despite correlative studies in these populations there has not been an adequate way to investigate the hemodynamic variables that contribute to cortical thinning. Asymptomatic, unilateral high grade internal carotid artery (ICA) stenosis may provide a model for one potential factor—altered cerebral blood flow—because intra-subject assessments can be made, comparing blood flow in the occluded vs non-occluded side, and carotid vs non-carotid territory.

We recently reported asymmetry in cortical thickness between the cortex supplied by a unilaterally occluded carotid artery and the cortex in the opposite hemisphere.[15] This finding prompted us to examine blood flow differences as a potential causal factor in cortical thinning. In the present study, using the same cohort, we investigated the relationship between cortical thinning and regional cortical blood flow (rCBF). We hypothesized that such a relationship in the setting of severe carotid atherosclerotic disease, with a known cause for compromised blood flow but without frank cerebral infarction, would provide new mechanistic support for a link between altered cerebral hemodynamics and loss of cortical thickness.

## Materials and methods

### Study participants

Twenty-nine patients, age 50–93, 20 male, 27 right-handed, with unilateral 80–100% ICA occlusion but no stroke were included in the study. Inclusion criteria were: ≥ 80% carotid stenosis (n = 15) or complete occlusion (n = 14), with < 40% stenosis in the contralateral ICA. Other inclusion criteria were: asymptomatic status or TIA-only, fluent in English, and able to give informed consent. Exclusion criteria included prior clinical stroke, diagnosis of dementia, history of head trauma with loss of consciousness, current substance abuse, major psychiatric disease, NYHA Stage 3/4 congestive heart disease, or contraindication to MRI. Presence of hypertension and diabetes were defined as the patient being on medications for that condition. All participants signed the consent form approved by the Institutional Review Board of Columbia University Medical Center. This institutional review board specifically approved this study.

#### Carotid artery stenosis

Carotid artery occlusive disease was assessed by carotid Doppler ultrasound in our IAC-accredited Neurovascular Ultrasound Laboratory at Columbia University Medical Center, and some individuals additionally underwent magnetic resonance angiography, or computed tomographic angiography. Degree of stenosis by Doppler was determined by flow velocities in the internal carotid artery (ICA) at the carotid bifurcation. Peak systolic velocities (PSV) >250cm/sec were classified as “high grade stenosis;” total occlusion was determined by visualization of the obstruction with B-mode Doppler, and quantification of lack of flow in the distal ICA bifurcation segment. Sixteen (55%) of the patients underwent additional testing by structural imaging, and all were concordant with the Doppler findings. The extracranial vertebral arteries were insonated as well. All patients also underwent transcranial Doppler ultrasound using a 2MHz probe at the temporal window to determine if there was blunting of the middle cerebral artery (MCA), as an indication that the proximal obstruction from the ICA stenosis/occlusion was hemodynamically significant. The posterior circulation was also insonated via the suboccipital window.

#### Magnetic image acquisition and processing

Imaging was performed on a 3T Philips Achieva scanner at Columbia University Medical Center. The following sequences were obtained on each participant: 1) A high-resolution 3D T1-weighted magnetization-prepared rapid gradient echo (MPRAGE) image was acquired with the following parameters: TE/TR = 3ms/6.7ms, voxel size = 0.9 x 0.9 x 0.9 mm^3^, 120 axial slices. This image was used to compute both the regional cortical thickness (rCT)[15] and to extract tissue volume data for partial volume effect correction (PVEc) of the arterial spin labeling (ASL) images.[[16, 17]] 2) Fluid-attenuated inversion recovery (FLAIR) images were acquired in the Multi-Slice Turbo Spin Echo (MS-TSE) mode with FOV = 250 mm, acquisition matrix of 192x133 resampled to 256x256 in reconstruction, slice thickness = 3 mm, TE/TR = 144 ms/5500 ms, inversion recovery delay = 1900 ms, and flip angle = 90 deg. An automated segmentation method was used to quantify total white matter hyperintensity volume for each hemisphere using the T1-weighted and FLAIR scans, applying random forests and support vector machines.[18] 3) a single-delay, background-suppressed pseudocontinuous arterial spin labeling (pCASL) images were acquired with the following parameters: TE/TR = 14ms/4500ms, flip angle = 90deg, labeling duration, LD = 1950 ms, initial post-labeling delay, PLD_0_ = 1200ms, slice-timing = 75ms, number of slices = 12, slice thickness = 8mm, in plane resolution = 3.5mm x 3.5 mm. The imaging volume was positioned such that the primary cortex was covered by slices 10 and 11, with effective PLD 2.0s and 2.1s, respectively. A 2D gradient echo EPI readout was used with background suppression pulses applied at 1680ms and 2760ms. For each patient, an average ASL CBF image was computed from a total of 60 control/label pairs as described below. The LD and PLD parameters were chosen based on arterial transit times (ATT) estimates in an aging population[19], as well as pilot data from the same population from our group.

For PVEc ASL preprocessing and gray matter CBF extraction, we used SPM12 software and in-house written MatLab codes as detailed in our previous work [20, 21]. Briefly, for each subject: (1) all EPI images were realigned to the first acquired images; (2) Gray matter (GM), white matter (WM), and cerebrospinal fluid (CSF) posterior probability images representing voxel tissue content (in %) were obtained from subject’s MPRAGE using SPM12’s segmentation algorithm (http://www.fil.ion.ucl.ac.uk/spm/software/spm12/); (3) The tissue probability masks were summed and thresholded (at total tissue volume sum of 30%) to compute a “brain tissue mask” that was subsequently applied to the MPRAGE image to exclude any non-brain tissue voxels. We refer to this image as “deskulled”; and (4) The deskulled MPRAGE and the posterior probability maps were co-registered to the average control EPI. This allowed uniform coregistration in M1 and V1.

For each patient, the PVEc algorithm estimates: 1) the magnetization values, *m*_*GM*_, *m*_*WM*_, *m*_*CSF*_ using subject’s mean control SE-EPI image; and 2) the ASL difference values, *dm*_*GM*_ and *dm*_*WM*_, using the (control-label) difference image. The PVEc algorithm was performed in each patient’s native space using a regression kernel of 7x7x1 voxels, following the procedure described in prior work. [20, 22].

CBF was computed following the recommendation of the consensus paper[23], using the following parameters using adjusted PLD to account for the inter-slice acquisition time, *PLDs* = (slice-number –1)·(70 ms) + 1200 ms; labeling efficiency = 0.70. [24] For the motor cortex (M1; Brodmann area 4)) in the frontal lobe, resulting in an average PLD ~1830ms. The CBF formula was applied to the *dm*_*GM*_*/m*_*GM*_ and *dm*_*WM*_*/m*_*WM*_, separately, and provided a uniform distribution of flow within gray matter ROIs. While a net CBF was computed for each patient, only the GM CBF data averaged over the M1 region and the visual cortex (V1; Brodmann area 17) was used for the statistical analysis.

Cortical Thickness measurement was performed on the Freesurfer platform[25], using volumetric tissue segmentation and inference of cortical structure from each patient’s MPRAGE image as described in our previous work.[15] Skull stripping was performed using the in-house thresholding method described above.

**Fig 1:**
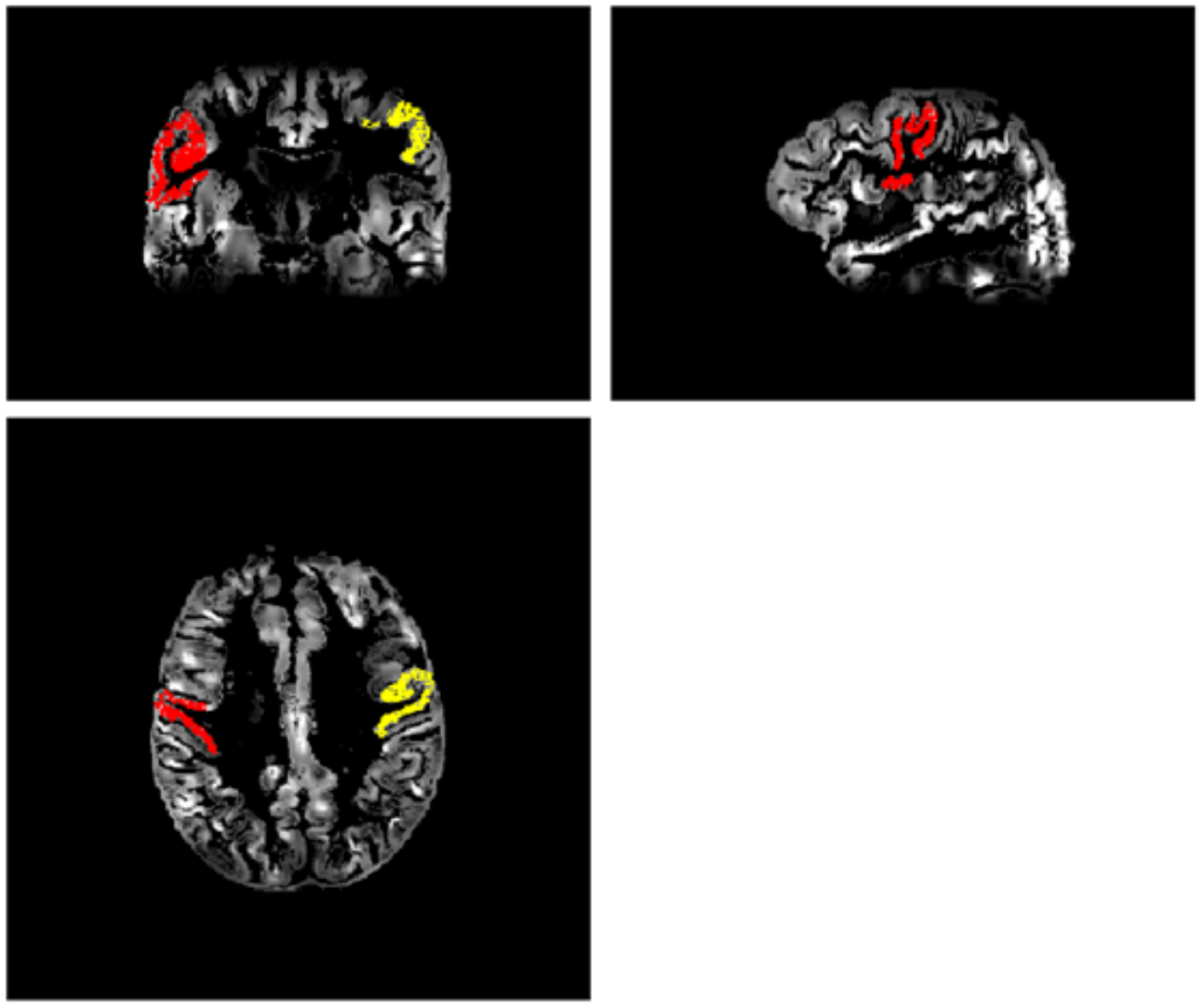
Cortical thickness-rCBF coregistration. A sample patient’s left (red) and right (yellow) motor cortex ROI, coregistered on the patient’s own GM CBF pCASL images. Images are shown in coronal, sagittal, and axial orientation.

ROI’s for an anterior circulation region—the primary motor cortex (M1: Brodmann Area 4)—, and a posterior circulation region—visual cortex (V1: Brodmann Area 17)—were extracted using the parcellation results. Once the ROIs were extracted from the Freesurfer space, the inverse coregistration matrix was used to bring the ROI-images back to the patient’s native space, i.e., co-registered with the pCASL CBF images. Specifically, the ASL images were upsampled and coregistered to the “deskulled” T1w image space. The co-registration of ASL to the cortical rim was done first manually and then using a newly developed SPM algorithm (CAT12) to ensure good quality co-registration. There was no significant difference in the number of voxels (volume of ROI) between the occluded and unoccluded side. An example of the left and right motor cortex ROIs co-registered to the motor cortex on the patient’s PCASL images is shown in Fig 1.

#### Statistical analyses

Paired *t*-tests were run to compare the GM CBF between the occluded and unoccluded sides in M1 and V1. Bonferroni adjustment was made for two comparisons (M1 and V1) for each measurement for the t-tests so that a two-sided P < 0.025 was required to declare statistical significance. Univariate Pearson correlation coefficients were calculated between cortical thickness in the two anatomical territories (M1, V1) and variables that were hypothesized to contribute to cortical thinning: age, resting gray matter CBF in M1 and V1 (M1 GM CBF, V1 GM CBF), hypertension, diabetes, and white matter hyperintensity volume (WMH). Univariate relationships were calculated for each hemisphere individually (occluded vs unoccluded). Variables with P-values ≤0.05 in the univariate analysis were entered into a multiple linear regression, using cortical thickness as the dependent variable. Occlusion versus high grade stenosis and circle of Willis collateral were also tested as independent variables in the regression. A generalized estimating equation (GEE) was also run with cortical thickness as the dependent variable with occluded vs unoccluded hemiepheral side as a covariate. Statistics were carried out using IBM SPSS Statistics version 23, and R version 3.1.2. Raw data are provided as supporting information (S1 Table).

## Results

**Fig 2:**
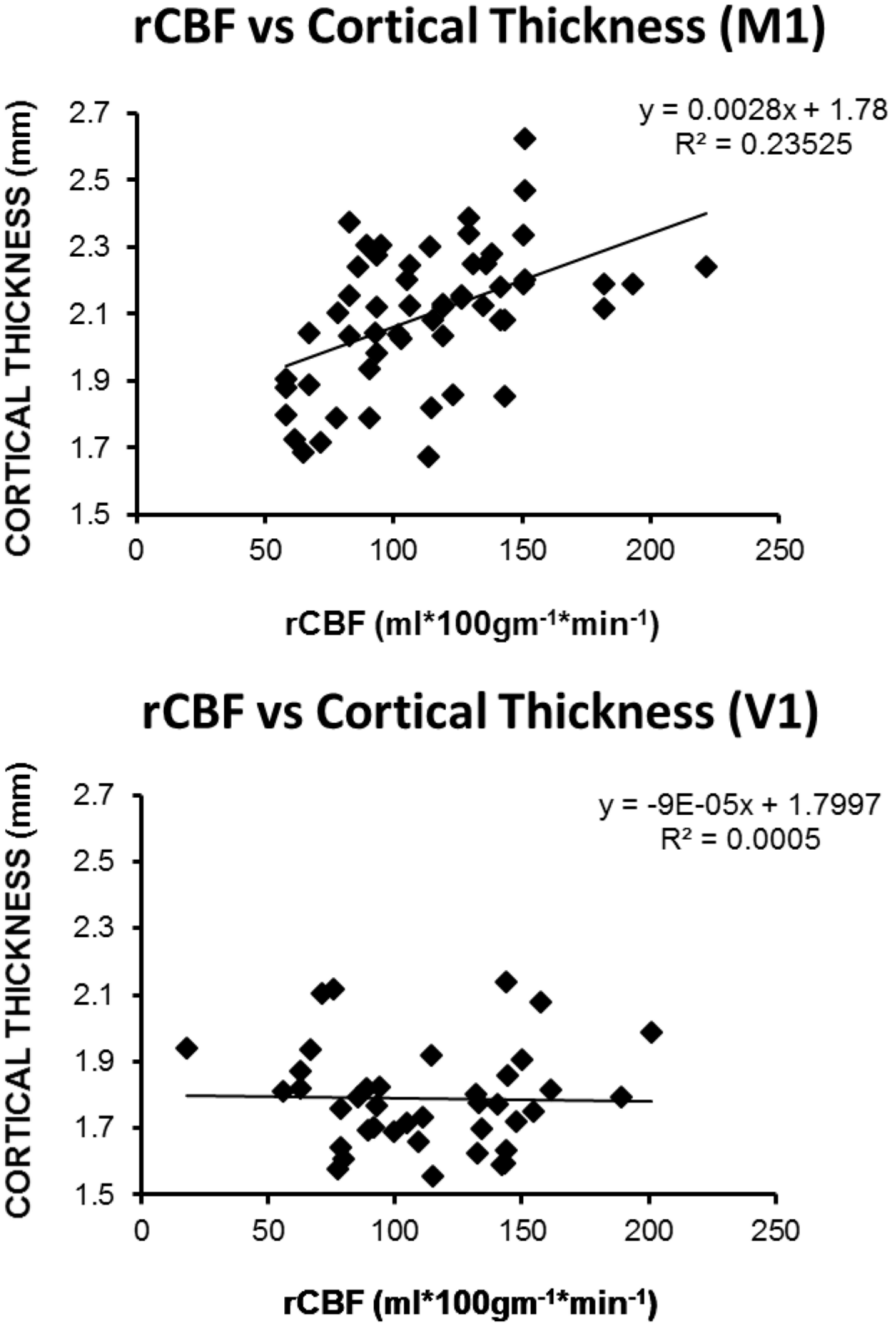
Scatterplots of cortical thickness versus CBF in primary motor cortex (M1, panel A) and in visual cortex (V1, panel B).

All 29 patients tolerated the MRI scanning protocol. One subject had ASL data that were uninterpretable, and this subject’s values were omitted from the analysis. Of the remaining 28, 14 had their occlusion on the left side, 14 had complete occlusion, 21 had hypertension, 6 had diabetes. There was no evidence for significant posterior circulation atherosclerosis, although there was mild basilar artery acceleration on transcranial Doppler in one patient, and in an extracranial vertebral artery in 4 patients by extracranial Doppler. Fifteen had evidence of cross-filling across the anterior circle of Willis, 6 had evidence of no cross filling, and in 7, the circle of Willis status was unknown. GM CBF was significantly lower on the occluded versus unoccluded side in the motor cortex (115.2 ml*100g^-1^*min^1^ vs. 105.5 ml*100g^-1^*min^-1^, P<0.0001) and in the visual cortex (112.8 vs. 106.4 P = 0.018). CBF asymmetry in the expected direction (lower on the occluded/stenotic side) was seen in 24 patients (86%), consistent with our previously published work.[26] In the GEE analysis, controlling for side of occlusion, circle of Willis collateral status, and occlusion vs stenosis, two variables—regional blood flow (0.0002) and age (p = .0020)—predicted cortical thickness in M1, but not in V1. Fig 2 shows a scatterplot of rCBF vs cortical thickness for M1 and V1, depicting the linear correlation between rCBF and rCT in M1, but not in V1.

Evaluating each hemisphere separately in the univariate analysis, we found that both age and regional blood flow correlated with cortical thickness for motor cortex in each hemisphere, respectively. None of the variables correlated with cortical thickness in the visual cortex. Table 1 shows univariate correlations with cortical thinning for the occluded and unoccluded side for motor and visual cortex. In the multiple linear regression analysis, regional blood flow predicted cortical thickness in M1 on both sides, age remained a predictor on the unoccluded side in M1, and there were no predictors of cortical thickness for V1 (Table 2). As reported previously[15], cortical thickness was significantly lower in the primary motor cortex on the side of carotid occlusion compared with the unoccluded side (2.07mm vs. 2.15mm, paired t-test, P<0.001), and no significant hemispheral asymmetry was present in the visual cortex (1.78mm on ipsilateral side vs. 1.80mm on contralateral side, paired t-test P>0.2).

**Table 1:** Univariate correlations with cortical thickness by side of occlusion.

	M1 Occl	M1 Unoccl	V1 Occl	V1 Unoccl
**Age**	**-.351 (0.045)**	**-.363 (0.038)**	-.011(0.950)	-.149(0.408)
**HTN**	-.325 (0.065)	-.278(0.117)	.068(0.708)	.036(0.844)
**DM**	-.131 (0.486)	.007(0.971)	-.012(0.949)	-.192(0.284)
**hCBF**	.359 (0.110)	.287 (0.208)	.135(0.521)	.076(0.720)
**rCBF**	**.494 (0.007)**	**.447(0.017)**	.093(0.689)	.218(0.343)
**hWMH**	.005 (0.980)	.080(0.697)	-.109(0.595)	-.299(0.138)

Numbers are Pearson correlation coefficient with univariate P-values in parentheses. M1 Occl = primary motor cortex on side of carotid occlusion, M1 Unoccl = primary motor cortex on side of normal carotid, V1 Occl = primary visual cortex on side of carotid occlusion, V1 Unoccl = primary visual cortex on side of normal carotid, HTN = HTN (0 = absent, 1 = present, on meds), DM = type 2 diabetes mellitus (0 = absent, 1 = present, on meds), hCBF = hemispheral cerebral blood flow (cortical gray matter by arterial spin labeling), rCBF = regional cerebral blood flow (cortical gray matter in cortical region listed), hWMH = white matter hyperintensity volume by hemisphere

**Table 2:** Multivariable regression by side of occlusion.

	M1 Occl	M1 Unoccl
**Age**	-.328(0.067)	**-.368 (0.039)**
**rCBF**	**.390(0.032)**	**.360(0.043)**

Numbers are Beta standardized coefficients with adjusted P-values in parentheses. M1 Occl = primary motor cortex on side of carotid occlusion, M1 Unoccl = primary motor cortex on side of normal carotid, V1 Occl = primary visual cortex on side of carotid occlusion, HTN = HTN (0 = absent, 1 = present, on meds), rCBF = regional cerebral blood flow (cortical gray matter in cortical region listed),

## Discussion

In our cohort of patients with asymptomatic high-grade carotid atherosclerotic disease without stroke, regional gray matter cerebral blood flow measured with pCASL correlated with cortical thickness in the motor cortex, bilaterally, but not in the visual cortex. In the anterior circulation, the motor cortex was thinner on the side of the occluded carotid artery where the blood flow was significantly lower, whereas no such asymmetry in cortical thickness was found in the visual cortex, despite a similar asymmetry in blood flow. Although not assessed directly, a fetal origin PCA, present in 15–30% of the population[27], could account for some of the flow asymmetry in the visual cortex. The degree and distribution of cortical thinning in the anterior circulation was, in fact, comparable to thinning that has been reported with unilateral chronic cerebral infarction,[28] in which motor cortex thickness in patients with a single underlying subcortical stroke was 2.07mm, identical to our M1 thickness on the side of carotid occlusion in the absence of stroke. On the contralateral side, their motor cortex measurement was 2.27mm, comparable to our average of 2.15mm on the side contralateral to the high-grade carotid stenosis. In that study, age-matched control subjects with no ischemic disease had an average M1 thickness of 2.40mm. In contrast to the motor cortex in the anterior circulation, our cohort’s average V1 thickness was 1.79 mm, similar to age-adjusted normal thickness in V1 of 1.81mm.[29] No patients in our cohort had frank stroke, and WMH volumes and silent subcortical infarcts did not correlate with cortical thinning. Taken together, our results extend our prior findings that cortical thickness differed by side of occlusion,[15] and suggest that there is a differential susceptibility to cortical thinning in the anterior circulation that was not present in the posterior circulation. Moreover, this susceptibility had both a generalized effect across both hemispheres in the carotid territories, as well as a hemispheral effect, based on presence of the unilateral high grade obstruction that produced hemispheral hypoperfusion. The thickness of the visual cortex, by contrast, does not appear to have been affected by blood flow, either in a generalized way, nor hemispherally.

Cortical thinning has been reported in cerebrovascular disease, but to date has only been associated with stroke risk factors such as hypertension and diabetes, or has been demonstrated in the setting of clinical or image-identified infarction. Three of the variables we measured have been previously reported to contribute to cortical thinning: age, diabetes, and hypertension. Hypertension was highly prevalent in our cohort, occurring in 75%, and diabetes was present in 21%, but neither was a significant predictor of cortical thickness in our analysis. Chronic hypertension in the general population has been reported to be associated with cortical thinning in the frontal and temporal lobes.[30, 31] Elevated blood glucose among patients with diabetes has been shown to be associated with cortical thinning,[10, 32, 33] and may additionally interact with hypertension.[11] Age, a widely reported correlate of cortical thickness[34–36], was associated with thinning in the motor cortex in our cohort, but not in the visual cortex, consistent with some reports suggesting that frontal, parietal, and temporal thinning are more closely associated with the aging process.[29, 37]. In patients with frank ischemic lesions, cortical thinning has been shown to be associated with subcortical stroke, silent microinfarcts and white matter hyperintensity burden[13, 14, 28]

Cortical thinning has been associated with reduced cerebral blood flow in a number of conditions, including Alzheimer’s disease. Lower CBF in the temporal and parietal regions of Alzheimer patients has been reported, but was thought to be reflective of reduced metabolism from tissue loss rather than being a causal factor, and low blood flow in frontal regions was postulated to be an effect of diaschisis.[38] In cerebrovascular and cardiovascular disease, hypoperfusion has been more commonly considered as a predictor variable for cortical thickness. It has been reported that among patients with hypertension and other cardiovascular risk factors, total brain perfusion predicted total brain volume and total brain cortical thickness, occurring predominantly in the frontal, temporal and parietal lobes bilaterally.[9, 39] In another study, cortical thickness was assessed in 35 patients with NYHA Functional Class II heart failure and low left ventricular ejection fraction, with cortical thinning found in the frontal, parietal, temporal, and occipital lobes bilaterally.[12] In our cohort, the choice of motor cortex as a target to examine the association between cortical thickness and blood flow allowed us to take advantage of the concept of the distal field or “watershed” territory of the occluded carotid artery, where carotid flow is most affected by high grade blockages.[40] This human disease model provided the unique opportunity to study an effect of unilateral hypoperfusion on cortical thickness, which appears to have been supported by our results.

We interpret our findings to suggest a differential impact of cerebral hemodynamics in the anterior circulation, which may be related to a predominance of atherosclerosis in the carotid arteries. Our patients had severe atherosclerosis in the carotid arteries and minimal atherosclerosis in the vertebrobasilar system. It is known that carotid atherosclerosis is associated with increased arterial stiffness[41], and has been associated with increased stroke risk[42], greater WMH volumes[43], larger diameters of intracranial vessels[44], and cognitive impairment[45]. It is postulated that pulsatile blood flow entering the cranium is less dampened through atherosclerotic carotid arteries, and thus will transmit pulse wave forces more directly to the intracranial vasculature including the capillary bed that perfuses the cortex. The impact of this blood flow on cortical thickness is a possible manifestation of this effect, and is consistent with reports of mostly anterior circulation territory cortical thinning in cerebrovascular disease populations, as cited above. The posterior circulation in our cohort would have had less pulsatility effect with lesser atherosclerosis in the vertebral arteries. As a second potential biomechanical effect, the “T-intersection” at the top of the basilar artery is thought to act as a physical baffle, taking the brunt of the pulsatile flow[46], and thus provide additional dampening of hemodynamic effects to the distal posterior vasculature, reducing the negative impact of pulsatile flow to the posterior cortex.

**Fig 3:**
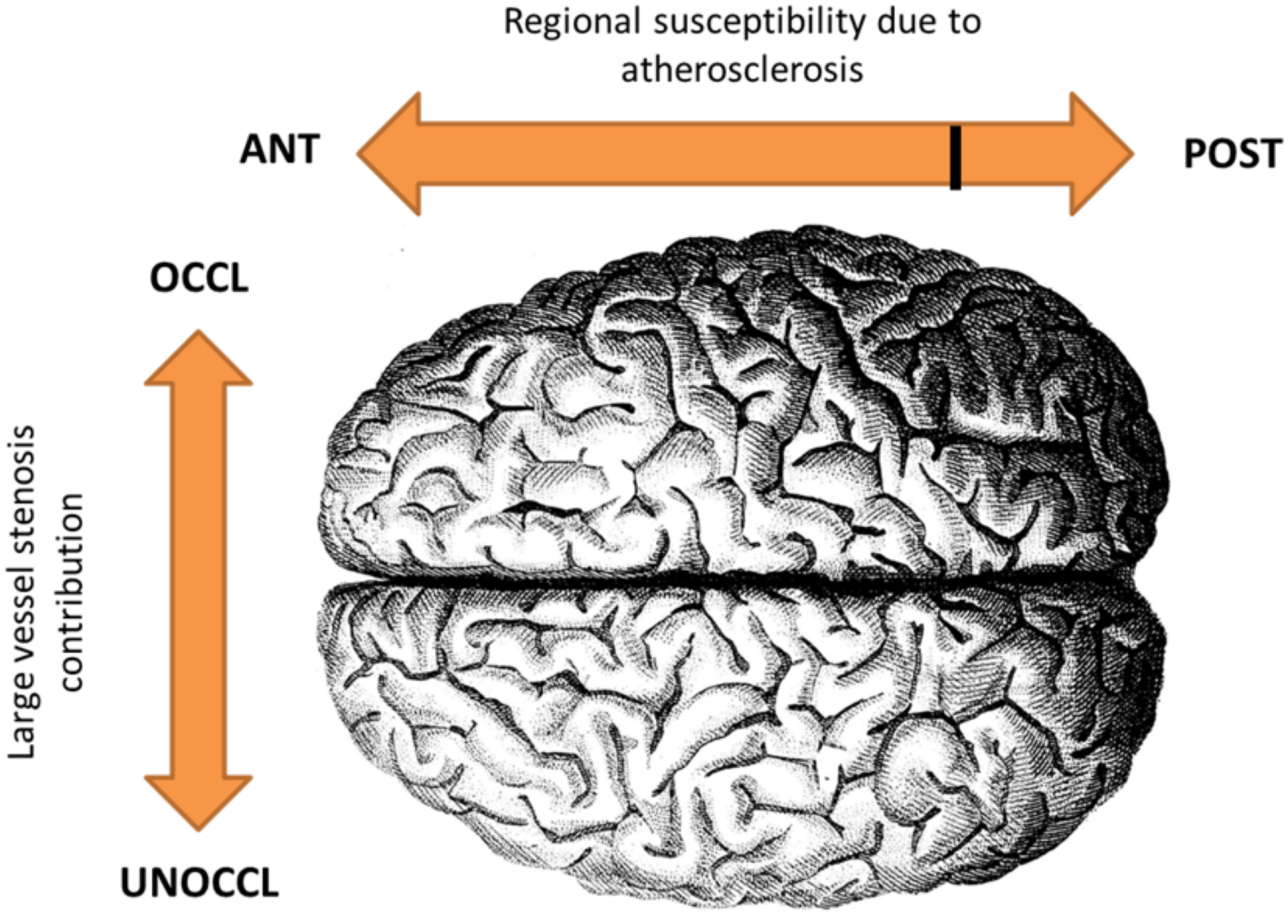
Two-factor model for the effect of altered hemodynamics on cortical thinning. We hypothesize a general susceptibility to thinning from atherosclerosis in the anterior circulation and a hemispheral effect of cortical thinning due to restricted flow from the high grade carotid stenosis.

In addition to the hypothesized anterior-posterior pulse flow differential, there was a hemispheral effect that resulted in greater cortical thinning on the side of the high-grade carotid stenosis where the resting cortical blood flow was significantly lower. Our hypothesis that altered hemodynamics is a contributor to cortical thinning would require that there exist a sufficient state of chronic or intermittent ischemia in the cortex, producing tissue loss in the absence of frank infarction. Although the pCASL rCBF measurements recorded in the M1 gray matter ranged from low into the high normal range, intermittent dipping into a hypoperfusion range may occur with sleep,[47] orthostatic hypotension[48, 49] or loss of autoregulation[50], exacerbating the impact of the abnormal hemodynamics. Cellular loss in chronic ischemic disease without infarction has been shown in a 3-vessel occlusion rat model in which CA1 neurons became damaged if the occlusion were left permanent, but remained functional if a reversal of the occlusion was performed within 2 weeks.[51] Histopathological findings in that study included reduced dendritic arborization and lower spine density that may change cortical architecture.[52] In humans, hemiatrophy has been shown in some patients with unilateral chronic ICA disease.[53] More recently, Positron Emission Tomography imaging with a benzodiazepine receptor ligand as a marker of neuronal integrity was used to identify selective neuronal loss (SNL) in 105 patients with high grade carotid stenosis but no cortical infarction on MRI. There was co-localization between SNL and increased oxygen extraction fraction, suggesting that areas of severe hypoperfusion may lead to tissue loss.[54] Overall, our findings of rCBF correlation with cortical thickness in both hemispheres in the anterior circulation, combined with the hemispheral asymmetry anteriorly, suggest that a two-factor hemodynamic effect may be contributing to cortical thinning—a generalized atherosclerotic effect, and a hemispheral effect due to hypoperfusion caused by the high grade unilateral stenosis. The two-factor conceptual model is illustrated in Fig 3.

We used pCASL to identify rCBF, although it has limitations. Due to scanning time constraints on the patients, we did not acquire simultaneous arterial transit time measurements on each patient. Instead, we opted for a relatively long labeling duration + post-labeling delay to include a wide range of ATTs expected in these patients. As per Alsop et al.[55], the maximum ATT accounted for in our study was 1.950 + 1.850 = 3.9s. This is well within the longest ATT measured by MacIntosh et al. in elderly subjects[19], as well as data acquired from a pilot study run by our group prior to this study in which the ATT in the ipsilateral side was on average ~1s longer than in the contralateral side We reiterate that while the PLD of this study is shorter than most ASL studies, we compensated by increasing the labeling durations to ensure that the SNR was not affected by the long PLD. As a result, there is risk for more vascular artifacts. Thus, the ATT remains an unmeasured factor in our study and the CBF results should be interpreted as a combined *true* flow plus transit time effect.

A second limitation is that with our method it is not possible to separate T1-w signal from gray matter versus blood, since they are similar at 3T.[56] This could potentially result in either an overestimation or an underestimation of cortical thickness. On one hand, since our patients are likely to have increased blood volume on the side of occlusion as is expected in “stage 1” hemodynamic failure,[57] there could be in an overestimation of cortical thickness. We demonstrated *thinner* cortex on that side, so this confound would support our findings. Conversely, there was, on average, lower CBF on the side of occlusion, which could result in an artifactual underestimation of cortical thickness. Since there was an asymmetry in cortical thickness in the motor cortex but not in the visual cortex despite an asymmetry in flow in both locations, however, it is unlikely that this potential confound was present. Although more research is needed to parse out these mediating effects, the overall effect would be expected to be less than the observed hemispheric difference in cortical thickness.

Limitations of this study also include its relatively small group size. A larger cohort might allow variables such as hypertension and diabetes to emerge as correlates of cortical thinning as reported in other studies. Another limitation is the lack of longitudinal data. We did not have follow up scans or information concerning duration of hypoperfusion, since patients entered the study with high grade carotid disease already present. The few patients who did not show a cortical thickness asymmetry in the expected direction may have had shorter total duration of hypoperfusion, or well-established collateral blood flow. Follow up imaging would help validate hypoperfusion as a cause of cortical thinning and document evolution over time. Our study was also limited by spatial resolution of the MRI scanner. Higher field scanning or concurrent metabolic imaging might elucidate anatomical features of the cortex that could give clues as to the nature of changes occurring in patients, such as selective neuronal loss, reduced synaptic complexity, or gliosis.

## Conclusion

We demonstrated in a cohort of patients with atherosclerotic high-grade carotid artery stenosis but no stroke that CBF was a significant predictor of cortical thickness. In addition to a general effect of rCBF correlating with cortical thickness in carotid territories, bilaterally, cortical thickness and CBF were significantly lower on the side of the higher carotid stenosis. In this patient population, we were able to take advantage of the carotid versus vertebrobasilar differential in atherosclerosis as well as a hemispheral asymmetry in degree of carotid stenosis to support this relationship. Because cortical thinning has been associated with cognitive impairment in several disease states, it is possible that cognitive impairment reported in high grade carotid artery disease[58, 59] may be driven by the hemodynamic effects of atherosclerotic arterial stiffness, and by chronic low cerebral blood flow on the side of high grade carotid stenosis, a notion which has support in animal models of chronic hypoperfusion. Further research is needed to assess cortical thickness longitudinally, and to determine the relative contributions of hypoperfusion and cortical thinning to cognitive impairment in these individuals.

## Supporting information

S1 Table Raw data. Raw data are listed in this table, each row representing a single subject. L_STEN = left stenosis, R_STEN = right stenosis, OCCL = occluded, XFIL = cross-filling, MFV = mean flow velocity, VMR = vasomotor reactivity, DCA = dynamic cerebral autoregulation, M1 = primary motor cortex, CBF = cerebral blood flow, V1 = primary visual cortex, thick = cortical thickness, WMH = white matter hyperintensity, HTN = hypertension, DM = diabetes mellitus.
(SAV)
